# Accuracy of glutamic acid decarboxylase antibodies for the identification of adult-onset type 1 diabetes mellitus: a systematic review and meta-analysis

**DOI:** 10.3389/fendo.2026.1771950

**Published:** 2026-03-31

**Authors:** Mandy M. Shao, Agatha F. Scheideman, Allen M. Zhou, Michael A. Kohn, Linda A. DiMeglio, Zoe Quandt, Cate Speake, Cindy N. Ho, David C. Klonoff

**Affiliations:** 1 Diabetes Technology Society, Burlingame, CA, United States; 2 University of California, Berkeley, Berkeley, CA, United States; 3 Department of Epidemiology and Biostatistics, University of California, San Francisco, San Francisco, CA, United States; 4 Division of Pediatric Endocrinology and Diabetology, Department of Pediatrics, Indiana University School of Medicine, Indianapolis, IN, United States; 5 Diabetes Center and Department of Medicine, University of California, San Francisco, San Francisco, CA, United States; 6 Benaroya Research Institute at Virginia Mason, Seattle, WA, United States; 7 Diabetes Research Institute, Mills-Peninsula Medical Center (Sutter Health), San Mateo, CA, United States

**Keywords:** autoantibodies, glutamic acid decarboxylase (GAD), sensitivity, type 1 diabetes (T1D), type 2 diabetes (T2D)

## Abstract

**Introduction:**

The glutamic acid decarboxylase autoantibody (GADA) test is a widely used marker to differentiate type 1 diabetes and type 2 diabetes in adults. We conducted a systematic review to estimate the sensitivity and specificity of the presence of GADA for T1D in adults with newly diagnosed diabetes.

**Methods:**

We conducted a PubMed and Embase search for studies that report both the GADA result and type 1 versus type 2 diabetes in adults diagnosed with diabetes. We calculated the sensitivity and specificity for each study.

**Results:**

We identified 19 studies involving 11,760 patients from diverse geographic settings. Across these studies, the sensitivity of GADA for identifying adult-onset type 1 diabetes varied widely (range: 0.27 to 0.83), with a pooled estimate of 0.53 (95% CI: 0.46–0.60). In contrast, specificity was consistently high, with a pooled estimate of 0.93 (95% CI: 0.89–0.96). The positive and negative likelihood ratios were 7.3 (95% CI 4.8 to 11.3) and 0.51 (0.44 to 0.58), respectively.

**Discussion:**

Our review demonstrates that GADA has high specificity and moderate sensitivity for identifying adult-onset type 1 diabetes. As a limitation, factors such as assay choice and cut-off values, as well as heterogeneity of both the type 1 and type 2 diabetes groups with regard to unmeasured genetic influences may contribute to the variability in antibody prevalence between studies. Our pooled likelihood ratios for GADA results might be useful for developing clinical and algorithmic tools to distinguish adult-onset type 1 diabetes from type 2 diabetes.

## Introduction

Adult-onset type 1 diabetes (T1D) is increasingly common, with symptoms that often overlap clinically with type 2 diabetes (T2D) (1). In an adult with new-onset diabetes, misclassification of T1D as T2D often leads to delays in initiation of insulin therapy and/or use of inappropriate treatments (2). More than 40% of adults with T1D over the age of 30 are initially misdiagnosed with T2D (3). Such misdiagnosis may lead to prolonged hyperglycemia and increased risk of ketoacidosis from delayed insulin therapy, highlighting the need for more reliable tools to support early differentiation of T1D from T2D in adults. Adult-onset diabetes poses unique diagnostic challenges because clinical features of autoimmune and non-autoimmune disease often overlap. In adults, features such as age at onset and body mass index may resemble T2D despite underlying autoimmunity, with genetic factors, including HLA variation further contributing to heterogeneity. This overlap contributes to frequent misclassification at diagnosis, increasing the risk of metabolic complications (4).

The detection of autoantibodies against pancreatic islet antigens is one of the primary methods of differentiating T1D from other forms of diabetes. The recent ADA Standards of Care emphasize the value of antibody testing in adults with newly diagnosed diabetes whose clinical features suggest autoimmune diabetes (5).

Among these autoantibodies, glutamic acid decarboxylase autoantibodies (GADA) are the most frequently measured in adults because of their high prevalence, persistence and clinical assay availability. Current clinical guidelines recommend GADA as the initial autoantibody test in adults with suspected autoimmune diabetes, with testing for islet tyrosine phosphatase 2 autoantibody (IA-2A) and zinc transporter 8 autoantibody (ZnT8A) when GADA is negative and the clinician still suspects autoimmune diabetes (5). This review focuses on GADA as the primary and most widely used autoantibody in diagnosing adult-onset T1D. Also, a preliminary search for studies of IA-2A identified only 5 studies for sensitivity and 2 studies for specificity. Similarly, a preliminary search for studies of ZnT8A identified only 5 studies for sensitivity and 3 studies for specificity.

Both the sensitivity and specificity of the GADA assay for diagnosing T1D are imperfect, which limit its clinical utility. Adults with new-onset T1D can be negative for GADA and those with T2D can be positive for GADA. Approximately 1 to 2 percent of the healthy population is GADA positive (6). An up-to-date analysis of GADA prevalence in T1D could be useful for investigators who are developing classification software tools to distinguish T1D from T2D in adults with new onset diabetes (7). To estimate the sensitivity and specificity of the presence of GADA for T1D in adults with newly-diagnosed T1D, we undertook a systematic review of diagnostic test accuracy (DTA) studies.

## Methods

### Data sources and search strategy

We followed PRISMA 2020 guidance (8). We searched PubMed from inception to 10 July 2025 using two complementary search strings for studies reporting GADA in adult T1D and T2D. The first query emphasized broad term coverage; the second added Title/Abstract field tags, adult/human filters, and diagnostic-accuracy terms. The first query returned 153 records and the second 136 records. One additional record was identified through reference-list screening, yielding 257 unique records for title/abstract screening. We also searched Embase from 1990 to 2025, which returned 417 records, of which 253 unique records remained after duplicate removal in Covidence. As a result of our search, we included 510 unique records for title/abstract screening. Full search strings appear in the **Supplementary Materials**.

### Eligibility criteria

We included studies that met the following criteria:

1. Reported data from individuals ≥ 18 years old living with diabetes;
2. Explicitly defined diagnostic criteria for classifying T1D and T2D;
3. Reported GADA positivity separately for T1D and T2D (or provided sufficient data to derive sensitivity and specificity)

We excluded case reports, pediatric-only studies, and studies lacking clear diagnostic definitions. Language and publication year were not exclusion criteria.

### Study selection

Three reviewers (M.A.K, M.M.S, and A.F.S) screened titles and abstracts in Covidence. Articles not excluded at this stage underwent full-text review against the same eligibility criteria by two reviewers (M.M.S and A.F.S). For records identified from Embase, title and abstract screening was performed by M.M.S. and C.H. Disagreements were resolved by discussion and consensus with a third reviewer (M.A.K).

### Data items and extraction (for included studies)

Data extraction was performed in Covidence using predefined extraction fields. To ensure consistency across studies with different diagnostic definitions and antibody cut-off thresholds, diagnostic criteria for T1D, T2D, and GADA positivity were extracted as reported verbatim in each manuscript. Any questions or disagreements arising during data extraction were resolved by discussion, with final determination by a third reviewer (M.A.K.) when needed.

We extracted: author, year, setting/country, definition of T1D, definition of GADA positivity, participant age, participant sex, and diagnostic data from each eligible study to construct 2×2 contingency tables for GADA positivity and negativity in their T1D and T2D populations. The four groups in the 2×2 tables were (1) True positive (TP) people with T1D who tested GAD-positive, (2) False negative (FN) people with T1D who tested GAD-negative, (3) True negative (TN) people with T2D who tested GAD-negative, (4) False positive (FP) people with T2D who tested GAD-positive. From these groups, we calculated the sensitivity, or the proportion of patients with T1D who were correctly identified as GAD-positive and the specificity, or the proportion of patients with T2D who were correctly identified as GAD-negative. The QUADAS-2 signaling questions were evaluated for all studies (9).

### Statistical analysis

We calculated sensitivity (Se) and specificity (Sp) with binomial 95% confidence intervals for each included study and across all studies. The overall estimates used the bivariate random-effects meta-analysis of logit(Se) and logit(Sp) implemented using the Stata metadta command, whose weighting accounts for the number of participants in each study. Results are displayed as forest plots of Se and Sp with 95% confidence intervals (CI) for each study and the pooled summary. Subgroup analysis was performed to investigate whether assay type (ELISA vs. radioimmunoassay) influenced diagnostic accuracy. The effect if assay type as a covariate in the bivariate random-effects model on pooled sensitivity and specificity was assessed using likelihood ratio tests comparing models with and without the covariate.

For each study we also computed the positive likelihood ratio (LR+) and negative likelihood ratio (LR-) with 95% confidence intervals. For studies with a zero cell in the 2×2 table, we added 0.5 to all four cells as a continuity correction when computing LRs. Summary LR+ and LR– were derived from the pooled Se and Sp estimated by the bivariate model. We assessed potential publication bias (small-study effects) using Deeks’ funnel plot asymmetry test for diagnostic test accuracy meta-analysis. This analysis was performed using the Stata MIDAS package.

## Results

**Figure 1:**
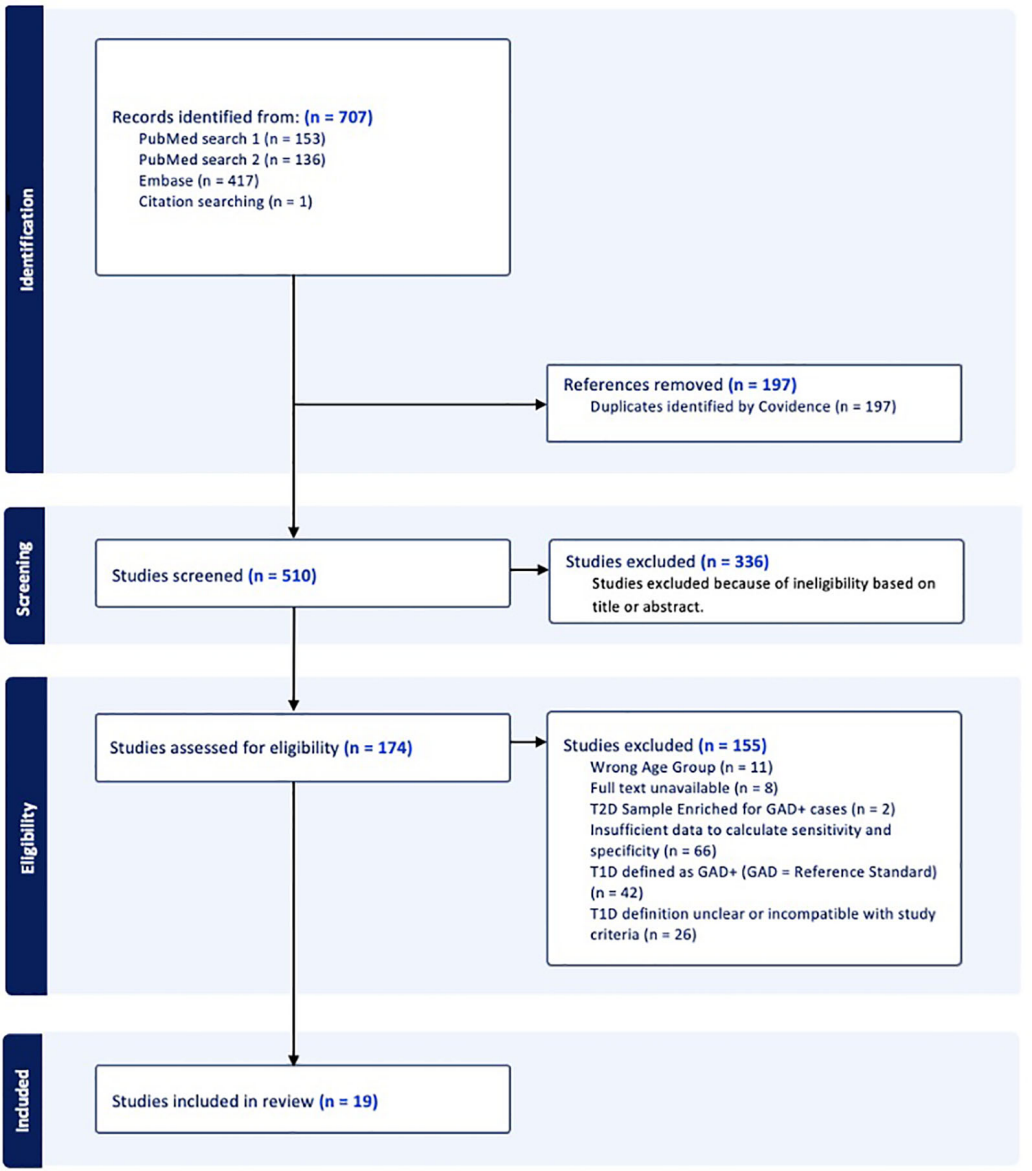
PRISMA 2020 flow diagram for systematic reviews using searches of databases and registers. Studies with diagnostic data for glutamic acid decarboxylase autoantibody (GADA) positivity and negativity in their type 1 diabetes (T1D) and type 2 diabetes (T2D) populations were selected in this process. Studies were included if they (1) reported data from individuals ≥ 18 years old living with diabetes; (2) explicitly defined diagnostic criteria for classifying T1D and T2D; and (3) reported GADA positivity separately for T1D and T2D (or provided sufficient data to derive sensitivity and specificity). Generated using Covidence.

Our literature search produced 510 unique publications. Of these, we reviewed the full text of 174 and ultimately identified 19 eligible studies encompassing 11,760 persons across North America, Europe, Asia, and Australia. The exclusion criteria are detailed in the PRISMA flow diagram (**Figure 1**).

The diagnostic definitions varied across studies, with some relying on clinical presentation and others incorporating insulin requirements and C-peptide levels. Overall, the study sizes ranged from fewer than 50 to more than 4,000 participants (**Table 1**).

**Table 1:** Summary of studies included in the systematic review of GADA sensitivity and specificity.

First author	Year	Country	Number	Proportion T1D	Definition of Type 1	Assay type	Definition of GAD+
Tuomilehto	1994	Finland	39	71.8%	Insulin therapy requirement	radiobinding/radioimmunoassay	>18 U
Zimmett	1994	Australia	65	29.2%	Insulin therapy requirement	radiobinding/radioimmunoassay	>18 U
Akamine	1997	Japan	66	27.3%	WHO criteria	radiobinding/radioimmunoassay	≥1 U/mL
Schiel	1998	Germany	189	50.3%	Expert Committee on the Diagnosis and Classification of Diabetes mellitus	radiobinding/radioimmunoassay	≥8 U/mL
Schranz	2000	Sweden	695	84.5%	WHO criteria (1992)	radiobinding/radioimmunoassay	ROC-derived cutoff index ≥ 0.05
Hawa	2000	Italy	252	20.6%	Insulin therapy from diagnosis, presence of diabetic ketoacidosis or marked ketonuria, absence of obesity, and onset after age 40 years	radiobinding/radioimmunoassay	>3 SD above control population
Kelly	2001	China	136	25.0%	Acute presentation of diabetes, with heavy ketonuria or ketoacidosis, and a requirement for continuous treatment with insulin from diagnosis	radiobinding/radioimmunoassay	>18 U
Barova	2004	Czech republic	259	68.0%	Insulin therapy requirement and low C-peptide levels	ELISA	>32 ng/mL
Ng	2009	Singapore	141	24.1%	Classical acute presentation with diabetic ketoacidosis at diagnosis or requirement for insulin at diagnosis and continued use for at least 1 year.	radiobinding/radioimmunoassay	>1.5 U/mL
Roh	2010	Korea	305	12.1%	Insulin-dependency within 6 months of diagnosis	radiobinding/radioimmunoassay	>1.47 U/mL
Zhang	2012	China	91	76.9%	Newly diagnosed lean (BMI <23 kg/m^2) adults (18–45 years) with low fasting C-peptide. Nearly all began insulin at diagnosis.	radiobinding/radioimmunoassay	≥5 U/mL
Fatima	2013	Pakistan	109	24.8%	Defined by clinical criteria (young onset, rapid insulin-dependent course, DKA, weight loss, absence of insulin-resistance features)	ELISA	≥10 U/mL
Dunseath	2015	United Kingdom	153	61.4%	Used American Diabetes Association criteria	ELISA	≥5 U/mL
Siraj	2016	Ethiopia	141	39.0%	T1D defined as failure to respond to oral agents and requiring insulin therapy	radiobinding/radioimmunoassay	>3 U/mL
Singh	2016	India	93	36.6%	Clinically diagnosed insulin-dependent diabetes	ELISA	> 5.0 U/mL
Wod	2017	Denmark	4374	11.5%	T1D defined as insulin-deficient diabetes (fasting C-peptide <300 pmol/L)	radiobinding/radioimmunoassay	≥25 U/mL
Luk	2019	China	1599	5.9%	Acute ketoacidosis or continuous insulin use within 1 year of diagnosis	ELISA	≥0.57 U/mL
Thomas	2019	UK	649	52.9%	Continuous insulin within 3 years of diagnosis and fasting C-peptide < 200pmol/L	ELISA	≥11 U/mL
Venkatesan	2024	India	2404	9.1%	Fasting C-peptide < 80 pmol/L	ELISA	≥10 U/mL

### Sensitivity and specificity

**Figure 2:**
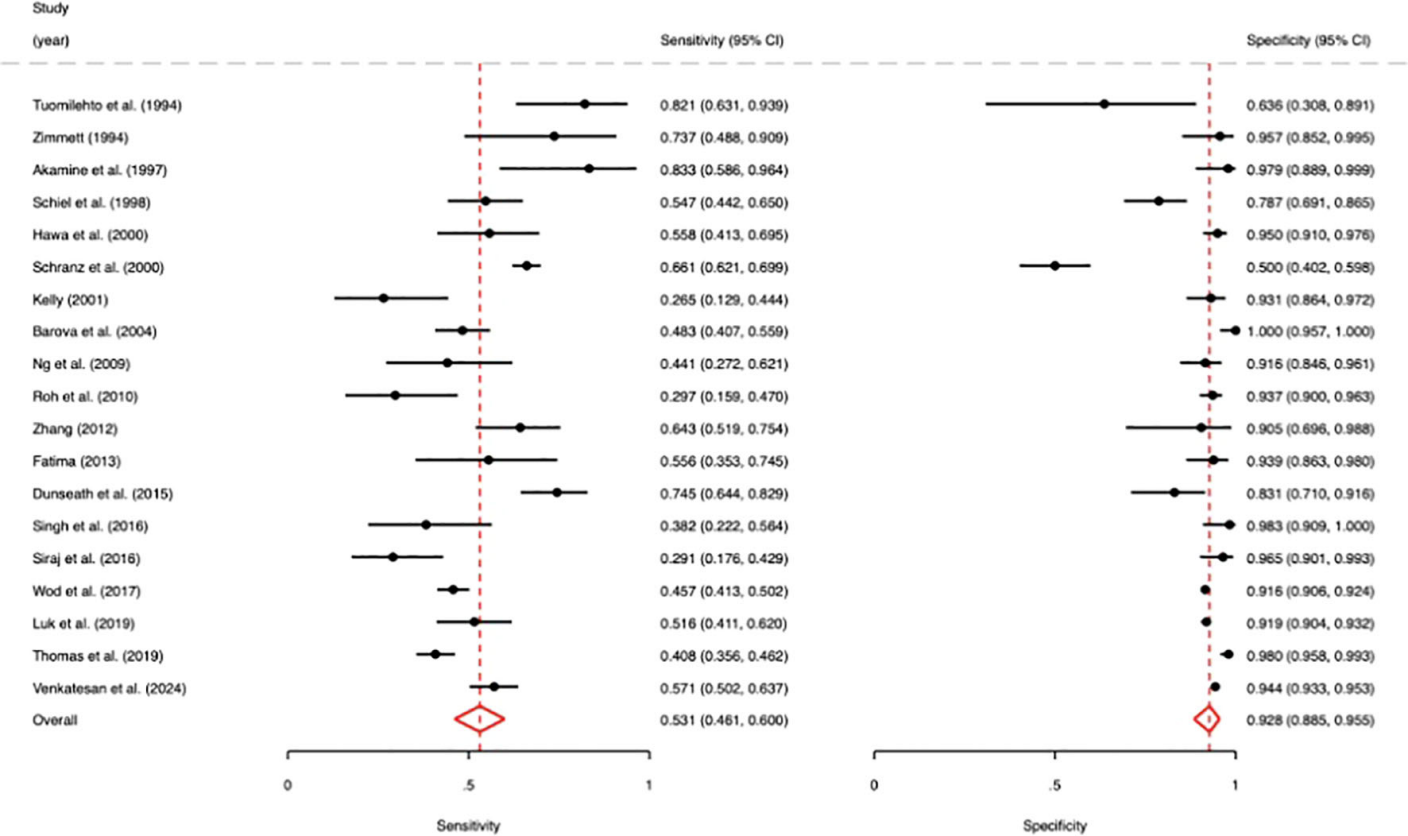
Forest plot of the sensitivity and specificity of glutamic acid decarboxylase antibodies (GADA) in diagnosing type 1 diabetes (T1D).

The reported sensitivity of GAD antibodies in identifying adult-onset T1D ranged from 0.27 to 0.83. The pooled estimate was 0.53 (95% CI: 0.46-0.60), which suggests that roughly half of people with adult-onset T1D tested positive for GADA. The sensitivities and specificities of each study are depicted in **Figure 2**. The specificity of GADA was consistently high across all included studies, with a pooled estimate of 0.93 (95% CI: 0.89-0.96) meaning that about 7 percent of patients with T2D are positive for GADA. Subgroup analysis by assay type showed no significant difference in sensitivity or specificity between ELISA and radioimmunoassay studies (sensitivity, p = 0.965; specificity p = 0.073).

The pooled LR+ and LR-, respectively, for GADA were 7.3 (95% CI 4.8 to 11.3) and 0.51 (0.44 to 0.58). These data indicate that people with adult-onset T1D are around seven times more likely to test positive for GADA than people with T2D while a negative GADA result reduces the odds of T1D by around one half. Deeks’ funnel plot asymmetry test showed no evidence of asymmetry (p = 0.51), suggesting no detectable small-study effects/publication bias. Ultimately, we excluded all studies with any QUADAS-2 signaling question with a domain that had unclear or high risk of bias.

## Discussion

In this systematic review and meta-analysis of 19 studies, we found that GADA demonstrated a pooled sensitivity of 0.53 and specificity of 0.93 for identifying T1D in adults. These estimates correspond to a positive likelihood ratio (LR+) of 7.34 and a negative likelihood ratio (LR−) of 0.51, indicating that a positive GADA result increases the probability of autoimmune diabetes, whereas a negative result does not reliably exclude it. These findings highlight GADA as a highly specific but only moderately sensitive marker for autoimmune diabetes in adults, with important implications for how antibody testing should be interpreted in clinical practice.

The specificity of 0.93 means that around 7 percent of adults classified as having T2D will test GADA positive. The moderate sensitivity of GADA in adults likely reflects biological heterogeneity in adult-onset diabetes. Genetic factors, including variation in HLA risk alleles, may contribute to heterogeneity and influence patterns of autoantibody expression in adults. Adults initially classified as having T2D who are GADA positive differ phenotypically from those who are GADA negative, suggesting an autoimmune component in some but not all cases. It remains unclear whether these individuals represent mild or slowly progressive autoimmune diabetes that is misclassified as T2D, or a distinct subtype in which GADA positivity reflects underlying pathophysiology without autoimmune destruction. Additionally, because other islet autoantibodies are not always assessed in clinical practice, reliance on GADA testing alone may underestimate autoimmune diabetes in adults because of low sensitivity.

GADA is one of four core autoantibodies typically used to diagnose T1D, including insulin autoantibodies (IAA), insulinoma-associated antigen 2 autoantibodies (IA-2A), and zinc transporter 8 autoantibodies (ZnT8A) (10). Adults with new-onset diabetes who have multiple islet autoantibodies are far more likely to have autoimmune T1D than adults who are positive for GAD antibodies alone (11). In an adult with new-onset diabetes, isolated GADA positivity supports but does not prove a diagnosis of autoimmune T1D. In an adult without clinical features of T1D, a single positive antibody does not prevent a diagnosis of T2D given the rate of GADA in T2D, which we found to be 7 percent. In clinical practice, when there is suspicion of autoimmune diabetes, testing for multiple autoantibodies remains essential.

People with T2D who test positive for GADA may be phenotypically distinct from those who are GADA negative. In the ADOPT trial, GADA-positive individuals had lower fasting insulin levels and higher insulin sensitivity than those who tested negative (12). Grubb et al. demonstrated that adults diagnosed with T2D who tested positive for GADA and carried a high T1D genetic risk score (GRS) progressed more rapidly to insulin therapy (13). Data from the RISE (14) and GRADE (15) trials further indicate that adults with T2D who are GADA positive frequently have T-cell reactivity to islet antigens. The degree of T-cell responsiveness, however, has variable association with residual insulin secretion (16). These findings suggest that autoimmune activity is present in some adults diagnosed with T2D and may meaningfully influence how these patients are characterized clinically. However, it remains unclear whether this reflects a true continuum of autoimmune diabetes across adult phenotypes or the coexistence of autoimmune diabetes with metabolic syndrome.

Emerging research aims to better define autoantibody negative T1D (17). In longitudinal cohorts, GAD autoantibodies (GADA) are generally more persistent and less likely to revert to negative than several other islet autoantibodies once an individual is multiple−autoantibody–positive (18). In individuals who are already multiple−autoantibody–positive, GADA is relatively stable and reversion to negativity is uncommon (18). The age of onset and duration of diabetes both have been reported to have an effect on antibody persistence (19). Most newly diagnosed T1D patients are GADA positive, but non−White groups (and especially some Asian and sub−Saharan African populations) have a lower prevalence of GADA positivity (20, 21).

### Strengths and limitations

To our knowledge, this is the first systematic review of GADA as a diagnostic test for T1D. A strength of this review is that it reports GADA antibody sensitivity and specificity from 19 studies in the world’s literature. Some factors such as assay choice and cut-off values may contribute to the variability in antibody prevalence between studies. Heterogeneity of both T1D and T2D groups with regard to Human Leukocyte Antigen (HLA) phenotype (particularly HLA-DR3) may also contribute to variability. Although HLA genotyping plays an important role in determining initial autoimmunity risk, this test is not generally used for predicting progression once autoantibodies develop. The American Diabetes Association recommends focusing on islet autoantibody testing rather than HLA typing for clinical decision-making in individuals with GADA positivity and new-onset diabetes (22). Programs that use standardized methods or participate in an autoantibody standardization program are more likely to generate reproducible results (11). However, participation in these programs was not an inclusion criterion in this review.

Although this analysis relies on traditional clinical classifications of adult-onset diabetes, which remain standard in practice, emerging research frameworks such as the Ahlqvist five-cluster model highlight underlying heterogeneity that may not be fully captured by binary T1D/T2D categories (23). In this context, some individuals classified here as GADA-negative T1D may reflect non-autoimmune insulin deficiency rather than true false-negative antibody testing.

Additionally, limited reporting on disease duration across studies is a limitation, as GADA prevalence is known to decline over time. Because GADA is generally more persistent than other islet autoantibodies, it may remain detectable at longer disease durations. However, the implications of this persistence for optimal testing timing are unclear, and while GADA testing may be informative at various time points, it is likely most useful when performed close to diagnosis.

## Conclusion

Across the studies in this review, we found that GADA is positive in 53 percent of adults newly diagnosed with T1D and in 7 percent of adults with T2D where T1D and T2D were differentiated by clinical criteria such as insulin dependence, C-peptide levels, and presence of ketoacidosis. GADA is a highly specific but only moderately sensitive marker of adult-onset T1D. We found that a positive GADA test increases the odds of T1D by about 7-fold (LR+ ≈ 7.3), whereas a negative test roughly halves the odds (LR− ≈ 0.51). Therefore, in new onset diabetes in an adult, a positive GAD test strongly supports the diagnosis of T1D, but a negative GAD test does not reliably rule out T1D. The variability in sensitivity across studies likely reflects differences in patient populations and different diagnostic definitions across populations.

Several academic groups and consortia are currently developing clinical and algorithmic tools to distinguish adult-onset T1D from type 2 diabetes at diagnosis (2, 24). Our pooled likelihood ratios for GADA results might be useful for these types of projects.

## References

[1] Aamodt KI Powers AC . The pathophysiology, presentation and classification of type 1 diabetes. Diabetes Obes Metab. (2025) 27:15–27. doi: 10.1111/dom.16628. PMID: 40734585 PMC12312824

[2] Leslie RD Evans-Molina C Freund-Brown J Buzzetti R Dabelea D Gillespie KM . Adult-onset type 1 diabetes: Current understanding and challenges. Diabetes Care. (2021) 44:2449–56. doi: 10.2337/dc21-0770. PMID: 34670785 PMC8546280

[3] Holt RIG DeVries JH Hess-Fischl A Hirsch IB Kirkman MS Klupa T . The management of type 1 diabetes in adults. A consensus report by the American Diabetes Association (ADA) and the European Association for the Study of Diabetes (EASD). Diabetologia. (2021) 64:2609–52. doi: 10.1007/s00125-021-05568-3. PMID: 34590174 PMC8481000

[4] Thomas NJ Jones AG . The challenges of identifying and studying type 1 diabetes in adults. Diabetologia. (2023) 66:2200–12. doi: 10.1007/s00125-023-06004-4. PMID: 37728732 PMC10628058

[5] American Diabetes Association Professional Practice Committee for Diabetes* . 2. Diagnosis and classification of diabetes: Standards of care in diabetes—2026. Diabetes Care. (2025) 49:S27–49. doi: 10.2337/dc26-S002. PMID: 41358893 PMC12690183

[6] Pihoker C Gilliam LK Hampe CS Lernmark Å . Autoantibodies in diabetes. Diabetes. (2005) 54:S52–61. doi: 10.2337/diabetes.54.suppl_2.S52. PMID: 16306341

[7] Cheheltani R King N Lee S North B Kovarik D Evans-Molina C . Predicting misdiagnosed adult-onset type 1 diabetes using machine learning. Diabetes Res Clin Pract. (2022) 191:110029. doi: 10.1016/j.diabres.2022.110029. PMID: 35940302 PMC10631495

[8] Page MJ McKenzie JE Bossuyt PM Boutron I Hoffmann TC Mulrow CD . The PRISMA 2020 statement: An updated guideline for reporting systematic reviews. BMJ. (2021) 372:n71. doi: 10.1136/bmj.n71. PMID: 33782057 PMC8005924

[9] Whiting PF Rutjes AW Westwood ME Mallett S Deeks JJ Reitsma JB . QUADAS-2: A revised tool for the quality assessment of diagnostic accuracy studies. Ann Intern Med. (2011) 155:529–36. doi: 10.1059/0003-4819-155-8-201110180-00009. PMID: 22007046

[10] Jia X Yu L . Understanding islet autoantibodies in prediction of type 1 diabetes. J Endocr Soc. (2023) 8:bvad160. doi: 10.1210/jendso/bvad160. PMID: 38169963 PMC10758755

[11] Felton JL Redondo MJ Oram RA Speake C Long SA Onengut-Gumuscu S . Islet autoantibodies as precision diagnostic tools to characterize heterogeneity in type 1 diabetes: A systematic review. Commun Med (Lond). (2024) 4:66. doi: 10.1038/s43856-024-00478-y. PMID: 38582818 PMC10998887

[12] Zinman B Kahn SE Haffner SM O’Neill MC Heise MA Freed MI . Phenotypic characteristics of GAD antibody-positive recently diagnosed patients with type 2 diabetes in North America and Europe. Diabetes. (2004) 53:3193–200. doi: 10.2337/diabetes.53.12.3193. PMID: 15561950

[13] Grubb AL McDonald TJ Rutters F Donnelly LA Hattersley AT Oram RA . A type 1 diabetes genetic risk score can identify patients with GAD65 autoantibody-positive type 2 diabetes who rapidly progress to insulin therapy. Diabetes Care. (2019) 42:208–14. doi: 10.2337/dc18-0431. PMID: 30352895 PMC6828553

[14] Brooks-Worrell BM Tjaden AH Edelstein SL Palomino B Utzschneider KM Arslanian S . Islet autoimmunity in adults with impaired glucose tolerance and recently diagnosed, treatment naïve type 2 diabetes in the Restoring Insulin SEcretion (RISE) Study. Front Immunol. (2021) 12:640251. doi: 10.3389/fimmu.2021.640251. PMID: 33981301 PMC8108986

[15] Brooks-Worrell B Hampe CS Hattery EG Palomino B Zangeneh SZ Utzschneider K . Islet autoimmunity is highly prevalent and associated with diminished β-cell function in patients with type 2 diabetes in the Grade Study. Diabetes. (2022) 71:1261–71. doi: 10.2337/db21-0590. PMID: 35061024 PMC9375448

[16] Hampe CS Shojaie A Brooks-Worrell B Dibay S Utzschneider K Kahn SE . GAD65Abs are not associated with beta-cell dysfunction in patients with T2D in the GRADE Study. J Endocr Soc. (2024) 8:bvad179. doi: 10.1210/jendso/bvad179. PMID: 38333889 PMC10853002

[17] Parikh HM Remedios CL Hampe CS Balasubramanyam A Fisher-Hoch SP Choi YJ . Data mining framework for discovering and clustering phenotypes of atypical diabetes. J Clin Endocrinol Metab. (2023) 108:834–46. doi: 10.1210/clinem/dgac632. PMID: 36314086 PMC10211492

[18] Vehik K Lynch KF Schatz DA Akolkar B Hagopian W Rewers M . Reversion of β-cell autoimmunity changes risk of type 1 diabetes: TEDDY Study. Diabetes Care. (2016) 39:1535–42. doi: 10.2337/dc16-0181. PMID: 27311490 PMC5001144

[19] Tridgell DM Spiekerman C Wang RS Greenbaum CJ . Interaction of onset and duration of diabetes on the percent of GAD and IA-2 antibody-positive subjects in the type 1 diabetes genetics consortium database. Diabetes Care. (2011) 34:988–93. doi: 10.2337/dc10-1903. PMID: 21330643 PMC3064062

[20] Siraj ES Gupta MK Yifter H Ahmed A Kebede T Reja A . Islet cell-associated autoantibodies in Ethiopians with diabetes mellitus. J Diabetes Complications. (2016) 30:1039–42. doi: 10.1016/j.jdiacomp.2016.05.005. PMID: 27220543

[21] Zimmet PZ Rowley MJ Mackay IR Knowles WJ Chen QY Chapman LH . The ethnic distribution of antibodies to glutamic acid decarboxylase: Presence and levels of insulin-dependent diabetes mellitus in Europid and Asian subjects. J Diabetes Complications. (1993) 7:1–7. doi: 10.1016/1056-8727(93)90016-r. PMID: 8481544

[22] Insel RA Dunne JL Atkinson MA Chiang JL Dabelea D Gottlieb PA . Staging presymptomatic type 1 diabetes: A scientific statement of JDRF, the Endocrine Society, and the American Diabetes Association. Diabetes Care. (2015) 38:1964–74. doi: 10.2337/dc15-1419. PMID: 26404926 PMC5321245

[23] Ahlqvist E Storm P Käräjämäki A Martinell M Dorkhan M Carlsson A . Novel subgroups of adult-onset diabetes and their association with outcomes: A data-driven cluster analysis of six variables. Lancet Diabetes Endocrinol. (2018) 6:361–9. doi: 10.1016/S2213-8587(18)30051-2. PMID: 29503172

[24] Lynam A McDonald T Hill A Dennis J Oram R Pearson E . Development and validation of multivariable clinical diagnostic models to identify type 1 diabetes requiring rapid insulin therapy in adults aged 18–50 years. BMJ Open. (2019) 9:e031586. doi: 10.1136/bmjopen-2019-031586. PMID: 31558459 PMC6773323

