# Supplementary material for: Accuracy of glutamic acid decarboxylase antibodies for the identification of adult-onset type 1 diabetes mellitus: a systematic review and meta-analysis
Source: Front Endocrinol (Lausanne). 2026 Mar 31;17:1771950. doi: 10.3389/fendo.2026.1771950 (PMC13076120; doi:10.3389/fendo.2026.1771950)
Supplement: Supplementary file 1 [file Supplementaryfile1.docx]

Supplement

Search String #1

(("Glutamic Acid Decarboxylase"[MeSH] OR GAD OR GAD65 OR "anti-GAD" OR "GAD antibody" OR GADA) AND ("Diabetes Mellitus, Type 1"[MeSH] OR "type 1 diabetes" OR T1D OR LADA OR "latent autoimmune diabetes") AND ("Diabetes Mellitus, Type 2"[MeSH] OR "type 2 diabetes" OR T2D) AND (prevalence OR sensitivity OR specificity OR diagnosis OR "case-control" OR "cross-sectional" OR cohort) ) NOT (pediatric OR child[MeSH] OR mice OR rat))

Search String #2

(("Glutamic Acid Decarboxylase"[Mesh] OR GAD65[tiab] OR GADA[tiab] OR "GAD antibody"[tiab] OR anti-GAD[tiab]) AND ( "Diabetes Mellitus, Type 1"[Mesh] OR "Diabetes Mellitus, Insulin-Dependent"[Mesh] OR T1D[tiab] OR IDDM[tiab] OR LADA[tiab] ) AND ( "Diabetes Mellitus, Type 2"[Mesh] OR "Diabetes Mellitus, Non-Insulin-Dependent"[Mesh] OR T2D[tiab] OR NIDDM[tiab] ) AND ( "Sensitivity and Specificity"[Mesh] OR sensitivity[tiab] OR specificity[tiab] OR prevalence[tiab] OR diagnostic[tiab] OR "diagnostic accuracy"[tiab] OR "cross-sectional"[tiab] OR cohort[tiab] ) AND ("Adult"[Mesh] OR adult[tiab]) AND "Humans"[Mesh] ) NOT ("Case Reports"[Publication Type]) NOT (mice[Mesh] OR rats[Mesh]))

Search String #3

('glutamic acid decarboxylase antibody'/exp/mj OR gad:ti,ab OR gad65:ti,ab OR gada:ti,ab OR 'anti-gad':ti,ab OR 'gad antibody':ti,ab OR 'glutamic acid decarboxylase':ti,ab) AND ('diabetes mellitus type 1'/exp/mj OR 'latent autoimmune diabetes in adults'/exp/mj OR ((('type 1' OR 'type i') NEAR/2 diabet*):ti,ab) OR t1d:ti,ab OR iddm:ti,ab OR lada:ti,ab OR (('latent autoimmune' NEAR/2 diabet*):ti,ab)) AND ('diabetes mellitus type 2'/exp/mj OR ((('type 2' OR 'type ii') NEAR/2 diabet*):ti,ab) OR t2d:ti,ab OR niddm:ti,ab OR (('non insulin dependent' NEAR/2 diabet*):ti,ab)) AND (classif*:ti,ab OR distinguish*:ti,ab OR differentiat*:ti,ab OR diagnos*:ti,ab OR sensitiv*:ti,ab OR specific*:ti,ab OR roc:ti,ab OR 'likelihood ratio*':ti,ab) AND 'article'/it AND [adult]/lim AND [humans]/lim AND [1990-2025]/py
